# Editorial: Legumes for global food security - volume II

**DOI:** 10.3389/fpls.2023.1273600

**Published:** 2023-09-18

**Authors:** Jose C. Jimenez-Lopez, Karam B. Singh, Alfonso Clemente, Jaroslaw Czubinski, Sergio Ochatt, Eric Von Wettberg, Petr Smýkal

**Affiliations:** ^1^ Department of Stress, Development and Signaling in Plants, Estación Experimental del Zaidín, Spanish National Research Council (CSIC), Granada, Spain; ^2^ Agriculture and Food, Commonwealth Scientific and Industrial Research Organization (CSIRO), Perth, WA, Australia; ^3^ Department of Nutrition and Sustainable Animal Production, Estación Experimental del Zaidin, Spanish National Research Council (CSIC), Granada, Spain; ^4^ Department of Biochemistry and Food Analysis, Poznan University of Life Sciences, Poznan, Poland; ^5^ Agroécologie, Institut National de Recherche pour l’Agriculture, l’alimentation et l’Environnement (INRAE), Institut Agro, University Bourgogne, University Bourgogne Franche-Comté, Dijon, France; ^6^ Department of Plant and Soil Science, University of Vermont, Burlington, VT, United States; ^7^ Department of Botany, Palacký University in Olomouc, Olomouc, Czechia

**Keywords:** legumes, global food security, crop improvement, nutrient management, sustainable agriculture, food and feed security, climate resilience

As the world faces an array of global challenges, including population growth, climate change, and the need for clean energy, the role of legumes in addressing these issues becomes increasingly significant. The Research Topic, *“Legumes for Global Food Security, Volume II”* aims to explore the diverse contributions of legumes in promoting sustainable agriculture and enhancing global food security.

Legumes play a crucial role in delivering vital services to societies worldwide. One of their primary contributions lies in their capacity to provide a wide range of food crops that serve as essential sources of plant-based proteins, addressing the challenge of food security for a rapidly growing population. Furthermore, grain legumes possess remarkable nutritional properties and act as cost-effective food choices, playing a pivotal role in achieving global food and feed security amid the growing world population. The significance of legumes extends beyond their role as a food source. Through rhizobial symbiosis, legumes have the unique ability to fix atmospheric nitrogen, enriching agro-ecosystems and boosting subsequent crop productivity by enhancing water and nutrient capture and recycling. Moreover, they play a key role in mitigating climate change, offering an alternative to synthetic nitrogenous fertilizers, which is energy-intensive to produce and release greenhouse gases upon breakdown. Furthermore, legumes contribute to the reduction of fossil fuel usage by providing biofuel feedstocks and industrial resources. Given the challenges posed by increasing climatic stresses, legumes’ genetic diversity equips them to thrive in various environments, rendering them resilient and ideal for sustainable intensification on small-scale and resource-constrained farms. Moreover, they play a vital role as biocontrol agents, effectively combating pests and diseases that could otherwise cause significant agricultural losses.

This Research Topic aims to explore the multifaceted contributions of legumes in the development of robust and efficient agro-ecosystems, thereby enhancing global food security. Here we summarize some of the highlights derived from the 22 articles published in this Research Topic, dividing them in four main topics, in order to better understand how research on legumes and related crops is contributing to crop improvement, adaptation, and nutrient management, ultimately aiming to address food security challenges and support sustainable agriculture.

Regarding crop adaptation to abiotic stress and improvement of abiotic stress tolerance, Gupta et al. assessed heat tolerance in urdbean genotypes to identify heat-tolerant cultivars suitable for cultivation during the summer season. In this paper, 97 diverse genotypes of urdbean (*Vigna mungo* L. Hepper) were assessed for yield under heat stress and non-stress conditions to identify heat-tolerant genotypes. Eight genotypes were highly heat tolerant, while 35 were highly heat sensitive. Physiological and biochemical characterization of selected genotypes under heat stress revealed variations in leaf nitrogen balance index, chlorophyll content, flavonols, and anthocyanin contents. Heat-tolerant genotypes exhibited higher membrane stability index and superior photosynthetic ability. Molecular characterization distinguished genetic differences between heat-tolerant (UPU 85-86) and heat-sensitive (PKGU 1) genotypes.

Then, Diaz et al. investigated the genetic relationship between iron and zinc concentration and yield in common beans to develop biofortified varieties with higher micronutrient levels. In their study, they focused on common bean (*Phaseolus vulgaris* L.), an important legume that provides cost-effective proteins and micronutrients, especially iron and zinc. To combat malnutrition in developing countries, biofortification aims to develop varieties with higher Fe/Zn content. However, breeders faced challenges due to the negative correlation between Fe/Zn concentration and yield. Using QTL mapping and GWAS analysis on biofortified parent populations, they identified 79 QTLs and 23 hotspot regions that showed QTLs with opposing effects on yield components and Fe/Zn accumulation. This allowed them to select specific QTLs to enhance Fe/Zn levels without compromising yield in biofortified cultivars.

Alkaloids, a diverse group of cyclic nitrogen-containing secondary metabolites, are present in over 20% of plant species, including *Lupinus albus* (white lupin), which naturally contains quinolizidine alkaloids (QAs) in seeds. While QAs provide natural protection, lupin-breeding programs have unintentionally selected against them due to limited understanding of the QA biosynthetic pathway. The review by Osorio and Till discusses the current state of research, focusing on natural mutations like the pauper locus and the use of molecular markers and sequencing technology to identify candidate genes. Precision breeding of low-alkaloid, high-nutrition white lupin can be achieved by understanding QA biosynthesis, essential for sustainable agricultural productivity and high-quality protein for food and feed.

Then, research performed by Woo Choi et al. aimed to improve soybean food quality by breeding a new soybean line with penta null recessive alleles for five antinutritional and allergenic components: lipoxygenase, KTI, lectin, 7S α′ subunit, and stachyose. The breeding resulted in a soybean strain with the penta null (lox1lox2lox3/lox1lox2lox3-ti/ti-le/le-cgy1/cgy1-rs2/rs2) genotype, devoid of the mentioned proteins and with low stachyose content. The new strain exhibited desirable agronomic traits, including purple flowers, tawny pubescence, determinate growth habit, and yellow pods at maturity. The 100-seed weight was 31.1 g, and the yield was 2.80 t/ha, making it a promising soybean line with improved nutritional attributes.

Finally, the paper by Li et al. focused on the important area of plant responses phosphate deficiencies. Phosphorus is a key micronutrient for crops and large areas of arable land (30-40%) are limited by phosphorus availability. This study identified *GmWRKY46* as crucial for enhanced phosphate starvation tolerance in soybean. It was induced in response to phosphate starvation, particularly in roots. Overexpression of *GmWRKY46* in transgenic Arabidopsis and soybean composite plants showed its involvement in root development, leading to increased phosphate uptake and improved growth under phosphate starvation. RNAseq and ChIP-qPCR analysis identified differentially expressed genes, including *AtED1*, which improved tolerance to phosphate starvation when overexpressed in transgenic Arabidopsis. *GmWRKY46* directly binds to a W box in the *AtED1* promoter. Overall, *GmWRKY46* represents a promising target for conventional breeding and transgenic approaches to enhance soybean’s phosphate starvation tolerance.

The second main theme of this Research Topic is genetic variability and yield improvement. In this regard, a study performed by Susmitha et al. assessed the genetic variability of grain nutrients in 600 pigeonpea germplasms from the RS Paroda Genebank, ICRISAT, India, aiming to identify potential sources for biofortification. Field trials in 2019 and 2020 revealed significant differences in agronomic traits and grain nutrients. Germplasms showed wide variation in days to 50% flowering, days to maturity, 100-seed weight, and grain yield per plant. Grain nutrients like protein, minerals (P, K, Ca, Mg, Fe, Zn, Mn, Cu) exhibited substantial variation. Germplasms from Asia had diverse grain nutrient levels, while those from Africa showed high nutrient density. Some germplasms exhibited favorable nutrient profiles and can serve as promising sources for developing biofortified lines with improved agronomic traits. The study’s phenotypic data can aid genetic improvement efforts through GWAS and SNP/haplotype-based approaches.

Then, Habtegebriel performed a study focused on genotype by environment interaction (GEI) in soybean, which affects breeding progress by hindering the selection of superior cultivars. The objective was to identify adapted and stable genotypes and explore potential mega-environments for future testing. Experiments were conducted over two years, evaluating yield components and other traits. Stability analysis using GGE biplot and AMMI model identified five genotypes as top performers. Genotypes JM-CLK/CRFD-15-SD (G8) and 5002T (G1) displayed the highest seed yield, making them suitable as parents for hybridization and commercial production. These genotypes are recommended for release as new soybean varieties for cultivation across various environments.

Rodas et al. investigated the *SUPERMAN* (*SUP*) gene, initially discovered in *Arabidopsis thaliana*, that maintains boundaries between reproductive organs, regulating stamen and carpel number in flowers. This study focuses on the *SUP* ortholog, *MtSUP*, in the legume *Medicago truncatula*. *M. truncatula* serves as a model to study unique developmental traits in legumes, like compound inflorescence and complex floral development. *MtSUP* plays a role in the genetic network controlling these processes, sharing conserved functions with *SUP* but also exhibiting context-specific novel functions in legumes. *MtSUP* controls flower number and determinacy of ephemeral meristems, providing insights into compound inflorescence and flower development in legumes. Understanding these genetic controls can benefit legume breeding for improved crop production and food security.

The interest in plant proteins for human and animal nutrition continues to grow with legume crops being at the forefront of efforts to breed crops with higher protein levels and better quality. The paper by Zhou et al. assessed two important protein quality traits in pea (*Pisum sativum* L.), a major grain legume grown widely worldwide. They used field trials spread over 3 years and in multiple locations to assess the amino acid profiles and protein digestibility in a pea recombinant inbred line (RIL) population consisting of 110 lines. While pea has reasonable levels of protein, it is low in some amino acids such as tryptophan, methionine and cysteine. The authors used near-infrared spectroscopy to measure the amino acid profiles in the RILs and showed that this method had advantages for such studies over other methods used previously, being a high-throughput, low cost and non-destructive method. The use of the method, coupled with the use of multiple sites and a three-year period, helped to identify QTLS associated with key amino acids as well as QTLS associated with protein digestibility. These QTLs and the improved method for measuring seed proteins will help with efforts to breed pea cultivars with improved nutritional qualities.

Given that their production in the tropics is located on marginal, rainfed lands and hence yield is low, Smith et al. assessed the adaptation of 12 bush bean (*Phaseolus vulgaris* L.) genotypes to individual and combined stress induced by drought and low P availability through their physiological and chemical responses. Thus, seed weight and aerial biomass were very significantly decreased by both stress factors, whether applied individually or in combination, and was coupled with an also decreased photosynthesis and stomatal conductance. Differences between genotypes were apparent though, and the common bean genotypes SEF60 and NCB226 were more resilient and better adapted to stress than the commercial control DOR390 providing a higher yield, but there were no statistically significant differences in the concentration of mineral nutrients and amino acids within the seed at harvest. Under water deficit, carbon assimilation and water use in leaves were reduced throughout development, while combined low P and water deficit resulted in significant changes in the concentration of key nutrients and amino acids in the soluble leaf fraction but did not impact the seed. Those results suggest that common bean genotypes have a certain degree of resilience in terms of yield as expressed through pod harvest index and conservation of seed nutritional content.

Keller et al. examined genetic variation for flowering time, yield, seed iron content, and growth habit in climbing common beans. In general, there has been paid less attention to the genetic basis of agronomic traits in climbing types than bush types of common bean, and the authors set out to correct this with an examination of 17 field trials and reanalysis of 16 previously published datasets from both the Meso-American and Andean genepools of common bean. In addition to finding a number of marker-trait associations, the authors show that genomic prediction is improved when bush bean information is included in models predicting climbing bean genotype-phenotype associations.

Finally, Zaki and Radwan studied the genotypic and phenotypic variance of five parental genotypes and six generated crosses in F1 and F2 generations. The results indicated a substantial degree of genotypic variability across the investigated variables, which was considerably greater than the phenotypic variance. The authors succeeded in obtaining crosses that possess promising potential for high yield.

The third topic includes five papers about improvement of nutritional quality of legumes. Salaria et al. reviewed the potential pathways for increase seed protein content, the role of anti-nutritional compounds, and the extent of genetic variation in cultivated lentil and its cross-compatible wild relatives. The authors discussed the need for careful phenotyping, and explored a range of breeding approaches considered as avenues for improvement including speed breeding, genomic selection, and genetic engineering.

To investigate the kinetics of iron (Fe) uptake and partitioning in chickpea, Jahan et al. used a hydroponic growth system and RNA-sequencing of six genotypes that vary in seed Fe content. A number of key transporters were found to be expressed in roots and leaves, with the genes *FRO2 and IRT1* being important in roots in the presence of Fe, and *GCN2* in low Fe conditions. Conversely, in leaves the genes *NRAMP3, V1T1, YSL1* along with storage gene *FER3* showed higher expression. The improved understanding of Fe dynamics provided by this work provides targets for efforts to increase chickpea seed Fe content under both high and low soil Fe conditions.

Carrillo-Pedromo et al. investigated cold tolerance in fava bean. They used two fava bean QTL mapping populations to investigate the genetic basis of cold tolerance. This investigation identified five genomic regions associated with improved overwintering tolerance. Investigation of synteny of these regions with the *Pisum* and *Medicago* genomes showed that these regions are also associated with cold tolerance across other closely related legumes, suggesting shared mechanisms of tolerance that could prove to be effective breeding targets.

Bautista-Expósito et al. analyzed the impact of germination on protein and phenolic compound profiles in lentil and fava bean seeds. In this paper, the authors decided to analyze how the profile of phenolic compounds in different seeds affects the duration of germination and digestibility of proteins. Hydrolysis of the main protein fractions (7S and 11S globulins) that occurred during germination resulted in a simultaneous increase in the content of peptides and free amino acids. In addition, the products of protein hydrolysis were tested by the authors for potential health-promoting properties, i.e., antihypertensive and antioxidant activities. The results of this study clearly showed that regardless of the type of seed, the germination process contributes to increase of protease activity and reduction of the level of phytic acid, trypsin inhibitors, and tannins which are considered antinutrients. Furthermore, the key role of seed permeability on the speed of the germination process, which subsequently influences the degree of antinutrients and the change in seed microstructure and endogenous proteolysis, was clearly demonstrated.

Finally, the work performed by Kalve et al. evaluated an interspecific population of chickpeas in terms of genome-associated stress tolerance indices. The approach used by the authors assumed the application of wild relatives as a source of novel alleles for adaptation to suboptimal environments. In particular, in their work, an interspecific population derived from *Cicer reticulatum* accessions was used as a donor of introgression of heat and drought tolerance. From the initial 600 interspecific lines that were generated and tested in terms of resistance to ascochyta blight, a 195-line subset was selected and studied. Subsequent analyses helped establish a set of individual lines that perform better under suboptimal conditions. The additional outcome of this research can be associated with identifying specific SNP markers that can be used as marker-assisted selection that can help identify genes underlying tolerance to abiotic stress.

The last five papers of this Research Topic were focused on the use of bio-inoculants and soil fertility improvement. Norris Savala et al. assessed the efficacy of bio-inoculants in soybean production in Mozambique. In this context, to improve soybean yield, farmers use bio-inoculants from various sources and agroecological adaptability. However, these bio-inoculants are often unavailable during planting time and vary in yield based on their source and handling. Mozambique relies on imported bio-inoculants from neighboring countries and even South America. In this study, seven *Bradyrhizobium diazoefficiens* strain-based bio-inoculants with different carrier materials were evaluated for their performance, adaptability, and soybean productivity. Inoculation significantly improved plant growth, nodulation, and yield, suggesting the potential of bio-inoculants to enhance soybean production in Mozambique.

Jacques et al. conducted two studies related to pea plant responses. The first study focused on the impact of mineral deprivation on nutrient content and remobilization. They imposed transient deprivation of 13 mineral nutrients during vegetative growth and observed preferential allocation of dry weight and elements to shoots, particularly tendrils. Different remobilization strategies were identified, and the study suggests strategies to enhance seed quality through precise fertilization during periods of mineral nutrient deficiency. The second study by Jacques et al. investigated pea plant responses to various water stress types and their effects on nutrient uptake and remobilization. Pea plants, being nutritionally important, face vulnerability to water deficits induced by climate change. Common responses to all water stress types were observed in shoots, with manganese (Mn) playing a significant role. Under continuous stress, boron (B) impacted root architecture. An “ecophysiological imprint” in the root system was also observed, leading to increased nodule numbers during the recovery period. These findings provide insights into plant strategies to cope with water stress and contribute to global food security and nutrient deficiencies while reducing reliance on animal products.

Agricultural soils are affected worldwide by acidic pH and high levels of aluminum (Al) contamination due to several factors including agriculture practices and climate change. Quinones et al. have reported the ability of lupin to tolerate and accumulate Al in the rhizosphere and inside the root cells, suggesting its use in the restoration of marginal acid Al-rich soils in temperate zones where other legumes are unable to grow. The authors describe several physiological and molecular mechanisms of tolerance, uptake and accumulation of Al by lupin; among them, cluster roots which are able to exudate organic acids anions and polyphenols as well as Al-tolerant rhizobia strains with ability to produce abundant exopolysaccharides. These adaptive mechanisms make lupin a suitable crop for acidic soils affected by Al toxicity.

Finally, Yu et al. identified the *GmTic110a* gene’s role in chloroplast development and its impact on soybean growth. More concretely, in this study, a Glycine max pale green leaf 3-1 (Gmpgl3-1) mutant with reduced chlorophyll content, chloroplast defects, decreased yields, and fewer pods per plant was isolated from soybean. Bulked segregant analysis and map-based cloning identified a mutation in the chloroplast development-related GmTic110a gene. Knockout plants exhibited similar phenotypes to the mutant. *GmTic110a* was highly expressed in leaves and localized to the inner chloroplast membrane. Interaction experiments revealed GmTic110a’s interaction with other proteins involved in chloroplast development (GmTic20, GmTic40a, and GmTic40b). These findings suggest the crucial role of *GmTic110a* in chloroplast development, impacting photosynthesis and soybean growth.

In conclusion, the Research Topic *“Legumes for Global Food Security, Volume II”* delves into the immense potential of legumes, paving the way for innovative and transformative solutions to address pressing 21st-century challenges. These 22 papers highlight the crucial role of legumes in providing essential plant-based proteins, supporting food and feed security in a cost-effective manner. Moreover, the diverse topics covered offer valuable insights to enhance crop improvement, adaptation, and nutrient management, ultimately contributing to global food security and sustainable agriculture. As a collective, these articles make a substantial contribution to understanding and breeding resilient legumes capable of withstanding climate challenges. Our hope is that these findings will significantly enhance global food security in the foreseeable future.

## Author contributions

JJ-L: Writing – original draft, Writing – review & editing. KS: Writing – original draft, Writing – review & editing. AC: Writing – original draft, Writing – review & editing. JC: Writing – original draft, Writing – review & editing. SO: Writing – original draft, Writing – review & editing. EW: Writing – original draft, Writing – review & editing. PS: Writing – original draft, Writing – review & editing.

